# Predicting the Risk of Microtia From Prenatal Factors: A Hospital-Based Case-Control Study

**DOI:** 10.3389/fped.2022.851872

**Published:** 2022-04-21

**Authors:** Wei Chen, Manqing Sun, Yue Zhang, Qun Zhang, Xiaolin Xu

**Affiliations:** ^1^Department of Plastic and Reconstructive Surgery, Shanghai Ninth People's Hospital, Shanghai Jiao Tong University School of Medicine, Shanghai, China; ^2^Department of Pediatrics, Ruijin Hospital Affiliated to Shanghai Jiao Tong University School of Medicine, Shanghai, China; ^3^Center for Clinical Big Data and Analytics, Second Affiliated Hospital and Department of Big Data in Health Science, School of Public Health, Zhejiang University School of Medicine, Hangzhou, China

**Keywords:** microtia, predictors, LASSO regression, conditional logistic regression, risk factors

## Abstract

**Background:**

Although a wide range of risk factors for microtia were identified, the limitation of these studies, however, is that risk factors were not estimated in comparison with one another or from different domains. Our study aimed to uncover which factors should be prioritized for the prevention and intervention of non-syndromic microtia via tranditonal and meachine-learning statistical methods.

**Methods:**

293 pairs of 1:1 matched non-syndromic microtia cases and controls who visited Shanghai Ninth People's Hospital were enrolled in the current study during 2017-2019. Thirty-nine risk factors across four domains were measured (i.e., parental sociodemographic characteristics, maternal pregnancy history, parental health conditions and lifestyles, and parental environmental and occupational exposures). Lasso regression model and multivariate conditional logistic regression model were performed to identify the leading predictors of microtia across the four domains. The area under the curve (AUC) was used to calculate the predictive probabilities.

**Results:**

Eight predictors were identified by the lasso regression, including abnormal pregnancy history, genital system infection, teratogenic drugs usage, folic acid supplementation, paternal chronic conditions history, parental exposure to indoor decoration, paternal occupational exposure to noise and maternal acute respiratory infection. The additional predictors identified by the multivariate conditional logistic regression model were maternal age and maternal occupational exposure to heavy metal. Predictors selected from the conditional logistic regression and lasso regression both yielded AUCs (95% CIs) of 0.83 (0.79–0.86).

**Conclusion:**

The findings from this study suggest some factors across multiple domains are key drivers of non-syndromic microtia regardless of the applied statistical methods. These factors could be used to generate hypotheses for further observational and clinical studies on microtia and guide the prevention and intervention strategies for microtia.

## Introduction

Microtia encompasses a spectrum of auricle malformation ranging from mild abnormalities in the contour and size of the ear to the complete absence of the ear (1). It could occur unilaterally or bilaterally, whereas most cases are unilateral (77% to 93%) and thus lead to asymmetries (2). The prevalence of microtia per 10,000 births worldwide ranges from 0.4 to 8.3 (2), and from 2.9 to 3.2 in China (3). Patients with microtia may suffer atresia external acoustic meatus and conductive hearing loss, which were associated with functional and psychosocial impairments (1, 4). Despite the developed auricular reconstruction for the treatment of microtia, long-term complications are prevalent, among which the cartilage framework resorption or distortion is mostly reported (5).

The pathogenesis and risk factors of microtia are poorly understood. Studies on predictors of microtia have identified a wide range of risk factors across multiple domains. Genetic components, including chromosomal abnormalities and genetic mutation, were confirmed to have significant effects on microtia (6, 7). However, most microtia cases are sporadic, suggesting the important role of non-genetic factors (4, 8, 9). For example, patients with microtia tended to be males and to have lower birth weights (10). Meanwhile, parental factors were also associated with microtia, such as advanced parental age, maternal use of medications, maternal acute illnesses and low maternal education (9–12). Moreover, maternal exposure to chemical substances and radiation during pregnancy in the workplace or home could increase the risk of microtia (13–15). Nonetheless, prior studies were limited to a few factors being estimated in isolation, making it difficult to infer which factors have the relatively stronger prediction.

Recent studies on microtia have moved beyond these siloed single-factors hypotheses, testing approaches to combine these independent predictors. For instance, Guo and colleagues have established a multivariate logistic model and identified seven significant factors of severe microtia (16). Similarly, a case-control study focused on severe microtia/atresia has spotted several environmental exposure factors using the multivariate logistic model (17). These studies, however, were limited to traditional statistical approaches, which could not be suitable to analyze a large number of variables simultaneously. Machine learning approaches could be suitable in this situation, and has been applied for diagnosis and risk prediction among many diseases (18, 19).

Our study, using data from hospital-based participants, aimed to identify the leading predictors of non-syndromic microtia from 39 risk factors across four domains (i.e., parental sociodemographic characteristics, maternal pregnancy history, parental basic conditions and lifestyles, and parental environmental and occupational exposures) through the comparison of traditional (multivariate conditional logistic regression) and machine-learning (lasso regression) models.

## Methods

### Study Population

In this hospital-based case-control study, subjects aged 0–10 were recruited from Shanghai Ninth People's Hospital, Shanghai Jiaotong University School of Medicine during 2017-2019. Children diagnosed with non-syndromic microtia according to Nagata's criteria were considered as cases (20), while those undergoing treatment for non-congenital diseases (e.g., injury and upper respiratory tract infection) in the same period were included in the control group. A 1:1 frequency matching was performed for cases and controls according to the same sex, the same region and age (<1 year). The exclusion criteria were: subjects with other major malformations (for example, polydactyly and syndactyly); inherited familial disease; family history of congenital deformities (including microtia) or hereditary diseases.

A total of 770 participants were initially enrolled. Thereafter, 184 participants were excluded due to the uncompleted questionnaire surveys or missing data on variates. In final, 586 eligible participants (293 cases and 293 controls) were included for further analysis.

### Data Collection

Basic information was collected through a detailed questionnaire, which included questions on characteristics of children and corresponding parents, as well as parental exposure to environmental and occupational factors during the periconceptional period. The periconceptional period was defined as 6 weeks before conception to the first trimester of pregnancy which is critical to embryonic development, including the formation of the auricle. A preliminary survey had been conducted to test the reliability and validity of the questionnaire.

### Measurements of Predictive Factors

Predictive factors were classified into four categories: parental sociodemographic characteristics, maternal pregnancy history, parental basic conditions and lifestyles, and parental environmental and occupational exposures. The residence of children was divided into urban and rural areas. Maternal pregnancy history consisted of gestational characteristics (i.e., parity, gravidity, and threatened abortion), abnormal pregnancy history, medication history (i.e., prenatal intake of ovulation stimulants drugs, teratogenic drugs, oral contraceptive and folic acid) and diseases history (i.e., genital system infection, urinary system infection, abdominal pain, vaginal bleeding, fever, acute respiratory infection and chronic diseases). Parental lifestyles contained parental smoking and drinking status during pregnancy, as well as maternal passive smoking status. Environmental factors included: industrial pollutants near residential areas (<3 km^2^), parental exposure to noise pollution, indoor decoration and new furniture. Parental occupational exposures were measured according to maternal exposure to noise, dust, radiation, heavy metal and organic solvent, and paternal exposure to noise, high temperature, dust, radiation, pesticides, insecticides, heavy metal and organic solvent.

### Statistical Analysis

Children with microtia were matched with controls at a rate of 1:1, according to the same sex, the same region and age (<1 year). Descriptive statistics (mean and standard deviation, or frequency and percentages) were used to summarize basic characteristics of participants and their corresponding parents using paired-samples *t*-test or chi-squared tests. Next, predictive factors were examined independently in the univariate conditional logistic regression model and ranked from strongest to weakest association with microtia according to odds ratios (ORs) and 95% confidence intervals (95% CIs). To identify the leading predictors of microtia, traditional (i.e., multivariate conditional logistic regression) and machine-learning (i.e., lasso regression) statistical approaches were performed. Based on the univariate analysis, factors with statistical significance were added as independent variables into the lasso regression model, with the cross-validation method conducted to identify the optimum model. Similarly, these factors were included in the multivariate conditional logistic regression model, with the stepwise selection method used to fit the model. Thereafter, two scores were created using the following formula based on the factors selected by these two models respectively: Score = β1^*^factor1 + β2^*^factor2 + β3^*^factor3+…, where β1, β2 and β3 denote the estimates of coefficients for factor 1, 2 and 3. The predictive probabilities of models were calculated by the area under the curve (AUC) according to these two scores. All analyses were performed using SAS (version 9.4, SAS Institute Inc.). All statistical tests were two-sided, and *P* < 0.05 was considered to be statistically significant.

## Results

### Basic Characteristics of Participants and Corresponding Parents

A total of 586 participants (293 cases and 293 controls) were enrolled in the current study. The basic characteristics of participants and their corresponding parents are summarized in Table 1. In terms of maternal characteristics, the mean maternal age at delivery was 27.5 ± 4.3 years old in the microtia group, older than its counterparts (26.3±4.1, *P* < 0.001). Moreover, mothers of children with microtia tended to have higher parity, gravidity and abnormal pregnancy history, undergo passive smoking, undergo threatened abortion, take medicines of teratogenic drugs and oral contraceptive during the prenatal period, have a lower rate of taking folic acid, suffer diseases (including genital system infection, urinary system infection, abdominal pain, vaginal bleeding, fever, acute respiratory or chronic diseases) and expose to heavy metal during pregnancy (*P* < 0.05). Fathers of children with microtia tended to be older, smokers, have chronic diseases and expose to occupational factors such as noise, dust, heavy metal and organic solvent (*P* < 0.05). For environmental factors, parents of children with microtia had a higher rate of exposure to industrial pollutants and indoor decoration (*P* < 0.05).

**Table 1:** Basic characteristics of participants and corresponding parents.

**Characteristics**	**Total (*N* = 586)**	**Controls (*n* = 293)**	**Cases (*n* = 293)**	***P* value**
**Children**				
Age (weeks) [mean (SD)]	7.7 (3.1)	7.7 (3.1)	7.7 (3.2)	0.903
Sex (%)				1.000
Male	432 (73.7)	216 (73.7)	216 (73.7)	
Female	154 (26.3)	77 (26.3)	77 (26.3)	
Region				1.000
Urban	386 (65.9)	193 (65.9)	193 (65.9)	
Rural	200 (34.1)	100 (34.1)	100 (34.1)	
**Corresponding mothers**				
Maternal age (years) [mean (SD)]	26.9 (4.1)	26.3 (3.7)	27.5 (4.3)	**<0.001**
Parity (%)				**<0.001**
1	424 (72.4)	231 (78.8)	193 (65.9)	
2	136 (23.2)	48 (16.4)	88 (30.0)	
≥3	26 (4.4)	14 (4.8)	12 (4.1)	
Gravidity (%)				**<0.001**
1	280 (47.8)	188 (64.2)	92 (31.4)	
2	161 (27.5)	66 (22.5)	95 (32.4)	
≥3	145 (24.7)	39 (13.3)	106 (36.2)	
Threatened abortion (%)				**0.002**
Yes	116 (19.8)	43 (14.7)	73 (24.9)	
No	470 (80.2)	250 (85.3)	220 (75.1)	
Abnormal pregnancy history (%)				**<0.001**
Yes	222 (37.9)	57 (19.5)	165 (56.3)	
No	364 (62.1)	236 (80.5)	128 (43.7)	
Prenatal intake of medicines				
Ovulation stimulants drugs (%)	26 (4.4)	12 (4.1)	14 (4.8)	0.688
Teratogenic drugs (%)	121 (20.7)	26 (8.9)	95 (32.4)	**<0.001**
Oral contraceptive (%)	44 (7.5)	14 (4.8)	30 (10.2)	**0.012**
Folic acid (%)	351 (59.9)	215 (61.3)	136 (46.4)	**<0.001**
Diseases history during pregnancy				
Genital system infection (%)	73 (12.5)	5 (1.7)	68 (23.2)	**<0.001**
Urinary system infection (%)	35 (6.0)	5 (1.7)	30 (10.2)	**<0.001**
Abdominal pain (%)	56 (9.6)	20 (6.8)	36 (12.3)	**0.025**
Vaginal bleeding (%)	113 (19.3)	38 (13.0)	75 (25.6)	**<0.001**
Fever (%)	55 (9.4)	18 (6.1)	37 (12.6)	**0.007**
Acute respiratory infection (%)	85 (14.5)	29 (9.9)	56 (19.1)	**0.002**
Chronic diseases (%)	58 (9.9)	19 (6.5)	39 (13.3)	**0.006**
Smoking status (%)				1.000
Yes	6 (1.0)	3 (1.0)	3 (1.0)	
No	580 (99.0)	290 (99.0)	290 (99.0)	
Passive smoking (%)				**0.003**
Yes	361 (61.6)	163 (55.6)	198 (67.6)	
No	225 (38.4)	130 (44.4)	95 (34.4)	
Drinking status (%)				0.145
Yes	41 (7.0)	25 (8.5)	16 (5.5)	
No	545 (93.0)	268 (91.5)	277 (94.5)	
Occupational exposure				
Noise (%)	23 (3.9)	9 (3.1)	14 (4.8)	0.288
Dust (%)	41 (7.0)	19 (6.5)	22 (7.5)	0.627
Radiation (%)	12 (2.1)	4 (1.4)	8 (2.7)	0.243
Heavy metal (%)	21 (3.6)	5 (1.7)	16 (5.5)	**0.015**
Organic solvent (%)	16 (2.7)	7 (2.4)	9 (3.1)	0.612
**Corresponding fathers**				
Paternal age (years) [mean (SD)]	28.8 (5.0)	28.2 (4.4)	29.5 (5.4)	**<0.001**
Smoking status (%)				**<0.001**
Yes	267 (45.6)	110 (37.5)	157 (53.6)	
No	319 (54.4)	183 (62.5)	136 (46.4)	
Drinking status (%)				0.738
Yes	340 (58.0)	173 (58.7)	168 (57.3)	
No	246 (42.0)	121 (49.2)	125 (42.7)	
Paternal chronic diseases (%)				**<0.001**
Yes	47 (8.0)	10 (3.4)	37 (12.6)	
No	539 (92.0)	283 (96.6)	256 (87.4)	
Occupational exposure				
Noise (%)	34 (5.8)	5 (1.7)	29 (9.9)	**<0.001**
High temperature (%)	17 (2.9)	11 (3.8)	6 (2.1)	0.218
Dust (%)	55 (9.4)	13 (4.4)	42 (14.3)	**<0.001**
Radiation (%)	8 (1.4)	3 (1.0)	5 (1.7)	0.477
Pesticides, insecticides (%)	5 (0.9)	2 (0.7)	3 (1.0)	0.653
Heavy metal (%)	53 9.0)	15 (5.1)	38 (13.0)	**<0.001**
Organic solvent (%)	33 (5.6)	11 (3.8)	22 (7.5)	**0.049**
**Parental exposure to environmental factors**				
Industrial pollutants near residential areas (<3 km^2^) (%)	115 (19.6)	44 (15.0)	71 (24.2)	**0.005**
Noise pollution (%)	93 (15.8)	41 (14.0)	52 (17.8)	0.214
Indoor decoration (%)	69 (11.8)	21 (7.2)	48 (16.4)	**<0.001**
New furniture (%)	88 (15.0)	41 (14.0)	47 (16.0)	0.488

*Values in bold indicate statistically significant*.

### Estimation of Microtia Risk for Each Independent Factor

**Figure 1:**
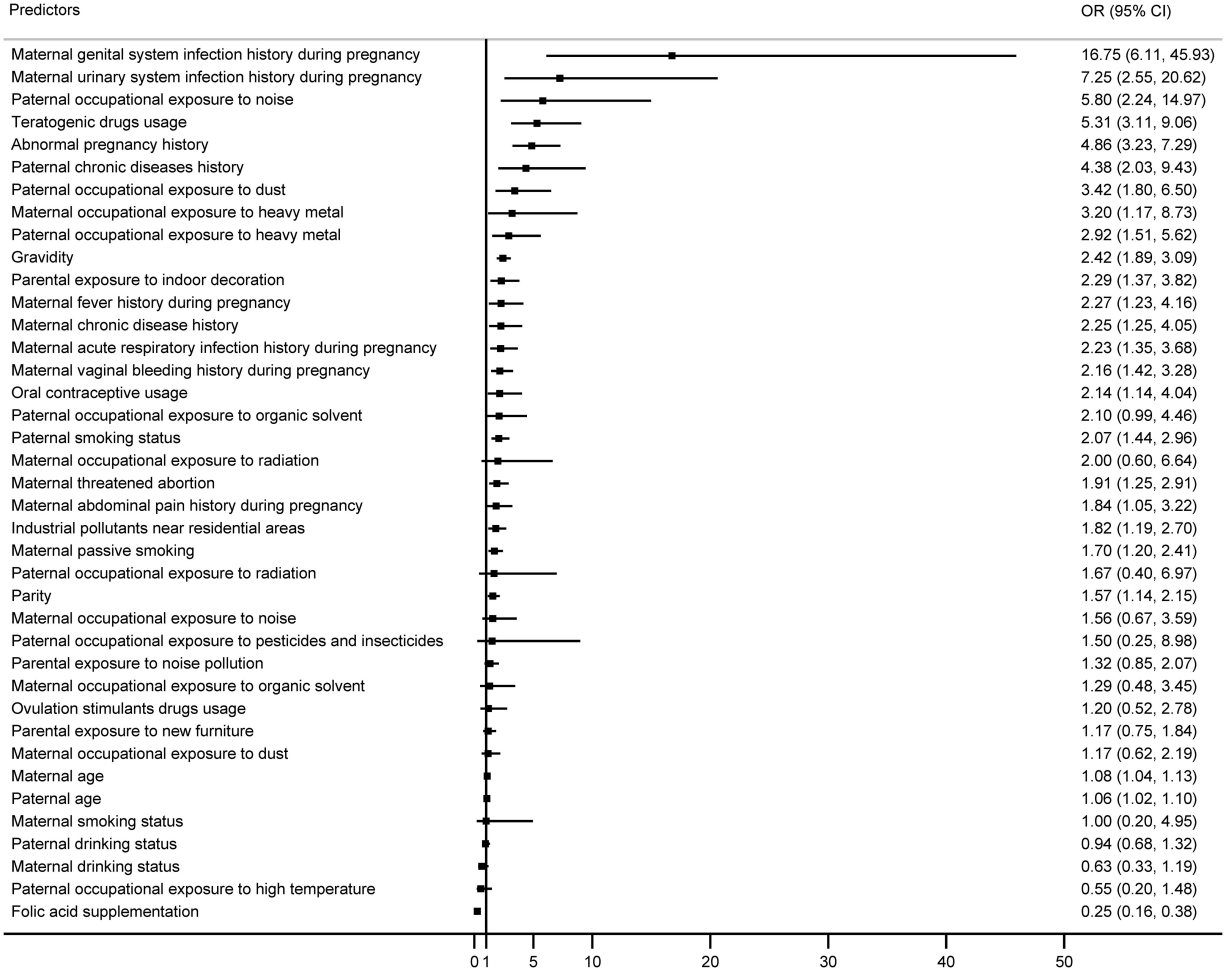
ORs and 95% CIs for the association between each predictor and microtia according to conditional logistic regression model.

The ORs (95% CIs) of each independent factor ranked from strongest to weakest association with microtia were shown in Figure 1. Of thirty-nine factors, twenty-five had confidence intervals that did not include 1. The top ten factors associated with higher risks of microtia were maternal genital (OR = 16.75, 95% CI = 6.11–45.93) and urinary system infection history (OR = 7.25, 95% CI = 2.55–20.62) during pregnancy, paternal occupational exposure to noise (OR = 5.80, 95% CI = 2.24–14.97), maternal intake of teratogenic drugs (OR = 5.31, 95% CI = 3.11–9.06), maternal abnormal pregnancy history (OR = 4.86, 95% CI = 3.23–7.29), paternal chronic diseases history (OR = 4.38, 95% CI = 2.03–9.43), paternal occupational exposure to dust (OR = 3.42, 85% CI = 1.80–6.50), maternal (OR = 3.20, 95% CI = 1.17–8.73) and paternal occupational exposure to heavy metal (OR = 2.92, 95% CI = 1.51–5.62), as well as maternal gravidity (OR = 2.27, 95% CI = 1.23–4.16).

### Predictors Selected by the Lasso Regression Model

We present in Table 2 predictors selected by the lasso regression model. The eight strongest factors were largely similar to the results from the independent conditional regression models. The additional predictive factors identified by the lasso model were folic acid supplementation, parental exposure to indoor decoration, and maternal acute respiratory infection during pregnancy, which were also strong predictors in the univariate conditional logistic models.

**Table 2:** Predictors selected by lasso regression.

**Predictors**	**Coefficients**
Abnormal pregnancy history	0.230
Genital system infection during pregnancy	0.213
Teratogenic drugs usage	0.151
Folic acid supplementation	−0.111
Paternal history of chronic conditions	0.061
Parental exposure to indoor decoration	0.031
Paternal occupational exposure to noise	0.024
Maternal acute respiratory infection during pregnancy	0.008

### Estimation of Microtia Risk Using the Multivariate Regression and Lasso Models

Predictive factors selected by the multivariate conditional logistic regression and lasso regression were compared in Table 3. In contrast to the results of the lasso regression model, paternal exposure to indoor decoration and maternal acute respiratory infection during pregnancy were not significant predictors according to the conditional logistic model, while maternal age and maternal occupational exposure to heavy metal were additional factors. Predictors selected from the conditional logistic regression and lasso regression both yielded AUCs (95% CIs) of 0.83 (0.79–0.86).

**Table 3:** The associations (ORs and 95% CIs) between selected predictors and microtia according to multivariate conditional logistic regression and lasso regression, respectively.

**Predictors**	**Conditional logistic regression**		**Lasso regression**	
	**OR (95% CI)**	***P* value**	**OR (95% CI)**	***P* value**
Maternal age	1.12 (1.05–1.20)	0.001	-	
Abnormal pregnancy history	3.93 (2.32–6.66)	<0.001	4.29 (2.56–7.18)	<0.001
Teratogenic drugs usage	4.19 (2.01–8.75)	<0.001	3.37 (1.62–6.97)	0.001
Folic acid supplementation	0.29 (0.16–0.53)	<0.001	0.33 (0.18–0.58)	<0.001
Genital system infection during pregnancy	14.75 (4.31–50.51)	<0.001	10.45 (3.20–34.10)	<0.001
Paternal history of chronic conditions	4.50 (1.48–13.62)	0.008	4.01 (1.45–11.10)	0.008
Maternal occupational exposure to heavy metal	4.99 (1.25–19.96)	0.023	-	
Paternal occupational exposure to noise	5.11 (1.32–19.80)	0.018	2.78 (0.80–9.70)	0.109
Maternal acute respiratory infection during pregnancy	-		1.70 (0.82–3.52)	0.153
Parental exposure to indoor decoration	-		2.04 (0.96–4.36)	0.065
AUC (95% CI)	0.83 (0.79–0.86)		0.83 (0.79–0.86)	

## Discussion

In our current study, thirty-nine factors across four domains were explored as independent predictors of microtia, and most of them were observed as important according to the univariate conditional logistic regression. Moreover, the leading predictors of microtia from different multivariate models varied. Although parental exposure to indoor decoration and maternal acute respiratory infection were strong predictors according to the lasso regression model, they reduced in strength when considered in the multivariate conditional logistic regression. Conversely, maternal age and maternal exposure to heavy metal were apparent in the multivariate conditional logistic regression, while they were not identified by the lasso regression model.

According to our results, the lasso regression model and multivariate conditional logistic model have identified eight leading predictors of microtia, respectively. These included factors across four domains: parental sociodemographic characteristics, maternal pregnancy history, parental basic conditions and lifestyles, and parental environmental and occupational exposures. The three most important predictors of microtia spotted by the lasso regression model were abnormal pregnancy history, genital system infection and teratogenic drugs usage, which remained significant in the multivariate conditional logistic regression model. Abnormal pregnancy history, such as pregnancy loss history, was a well-established risk factor of pregnancy outcomes in subsequent pregnancies (21). This finding informed us of the importance of antenatal surveillance and treatment targeted at women with a history of abnormal pregnancy. Moreover, the presence of ureaplasma urealyticum and mycoplasma hominis, along with bacterial vaginosis were reported to link with adverse pregnancy outcomes (22), consisting with the finding that genital system infection was associated with a higher risk of microtia. This evidence showed the whole vaginal microbiota is needed to investigate for understanding the etiology of microtia. Furthermore, teratogenic drugs, including antiepileptics and hydroxyethylrutosidea, were significantly associated with a higher risk of microtia according to previous research (23). Thus, the usage of teratogenic drugs should be contraindicated in pregnant women, especially in the second and third months of pregnancy.

Additionally, parental environmental and occupational exposures were illustrated as significant predictors, especially for indoor decoration, noise and heavy metal. With the urbanization and industrial development in China, women are undertaking diverse types of jobs. Accordingly, they may have more opportunities to expose to environmental and occupational pollutions, contributing to the increasing risks of environmental/occupational-related diseases and even affecting offspring health (24). Hence, quantifying the addictive effect of these factors could guide health promotion in pregnant women.

Inversely, maternal folic acid supplementation during pregnancy was spotted as the most important protective factor against microtia, consistent with previous findings (25, 26). As the critical role of folic acid in cell proliferation, it is indispensable during the period of fetal development. However, there were a significant number of pregnant women that did not have folic acid supplementation in China, especially in areas with relatively poor economics (27). Thus, the promotion of folic acid supplementation among pregnant women was still a priority of maternal and child health services. Regular monitoring and expanding surveillance time quantum are crucial for the prevention of birth defects such as microtia.

Despite the inconsistency, six factors were selected by the two models simultaneously, and these factors should be considered primarily. Moreover, the utility of different approaches is the identification of multiple predictors from across domains, which can expand future considerations of what types of predictors should be thoroughly investigated, and promote hypothesis generation for future research. Future studies focus on these predictors with large sample sizes are warranted for validation. Additionally, these findings would widen the net of crucial factors for the prevention and intervention of microtia by taking multiple predictors from across domains into consideration.

There remained some limitations in our study. First, although we ranked which factors best predict microtia from across disciplines, the case-control study design does not allow for causal interpretations. Second, our measures of microtia-related factors were limited in scope and number, for example, genetic factors could not be measured since we did not make genetic testing. These relevant risk factors should be considered in future studies. Third, factors involved in our study were collected based on self-report information, which would result in reporting and recall bias. Forth, we have not considered the grade of microtia severity in our current study, future studies with microtia severity are warranted. Last, the relatively small sample size of our study may lead to the instability of models, further studies are warranted for validation.

## Conclusion

Our study demonstrated that, in addition to well-established factors (e.g., sociodemographic characteristics, maternal pregnancy history, and parental basic conditions and lifestyles), parental environmental and occupational exposures were also among the strongest predictors of microtia. The strength of these predictors identified by different models could expand future consideration of which factors should be investigated primarily and provide information for transdisciplinary prevention and intervention of microtia.

## Data Availability Statement

The raw data supporting the conclusions of this article will be made available by the authors, without undue reservation.

## Ethics Statement

The studies involving human participants were reviewed and approved by the CAES (China Association For Ethical Studies) and was approved by the Ethics Committee of the Ninth People's Hospital in Shanghai. Written informed consent to participate in this study was provided by the participants' legal guardian/next of kin.

## Author Contributions

WC, MS, and YZ contributed to the design, data collection, data analysis of this study, and drafting the manuscript. QZ and XX contributed to the design, conceptualization, editing and revising the manuscript, and giving the final approval as submitted. All authors contributed to the article and approved the submitted version.

## Funding

The work were supported by grant to QZ from the National Natural Science Foundation of China (81372081) and grant to XX from China Medical Board Open Competition Program (21-416).

## Conflict of Interest

The authors declare that the research was conducted in the absence of any commercial or financial relationships that could be construed as a potential conflict of interest.

## Publisher's Note

All claims expressed in this article are solely those of the authors and do not necessarily represent those of their affiliated organizations, or those of the publisher, the editors and the reviewers. Any product that may be evaluated in this article, or claim that may be made by its manufacturer, is not guaranteed or endorsed by the publisher.

## Abbreviations

AUC: the area under the curve
OR: odds ratio
95% CI: 95% confidence intervals.
